# Steroids—has the time come to extend their use to AML?

**DOI:** 10.1186/s43046-021-00062-8

**Published:** 2021-03-04

**Authors:** Mariah Farrugia, Catriona Cutajar, Jean Calleja Agius, Pierre Schembri Wismayer

**Affiliations:** Department of Anatomy, Faculty of Medicine and Surgery, University of Malta, Msida, MDS2080 Malta

**Keywords:** Leukaemia, Steroids, Therapy, CNS, Ecdysteroids, Glucocorticoids, Cardiotonic steroids

## Abstract

**Background:**

In 2018, leukaemia accounted for 2.6% of all new cancers, it being the 13th most common cause of cancer and the 10th most common cause of cancer death. Glucocorticoids are commonly used in lymphoid leukaemia treatment, where they are cytotoxic. The aim of this review is to highlight ongoing research of steroid use in myeloid leukaemias.

**Main text:**

Glucocorticoids increase infection risks in acute myeloid leukaemia, but with adequate antifungal cover, they can help in hyperleucocytic disease. They also show some benefits in sensitising multidrug-resistant AML cell lines to cytotoxic agents, induce differentiation marker expression and can also induce CD38 expression, making AML cells possible targets of daratumumab.

Cardiotonic steroids, like digitalis, are being recognised as sensitising AML cells to the chemotherapeutic effects of many cytotoxic agents, primarily by inhibiting efflux pumps, thus minimising AML resistance.

Ecdysteroids enhance sensitivity in multidrug-resistant AML, but also in non-resistant AML cell lines, through pathways including the activation of mitochondrial apoptosis. Their anti-apoptotic effects on non-malignant cell lines help their target specificity. Sensitisation is chemotherapy-specific, enhancing the effects of doxorubicin and tubulin inhibitors but increasing resistance to cisplatinum.

**Short conclusion:**

Cardiotonic steroids and ecdysteroids both show chemosensitisation to the cytotoxic effects of chemotherapy on AML cell lines. It is likely time to consider clinical trials to assess whether these, as well as traditional glucocorticoids, can contribute to the AML armamentarium, particularly in chemo-resistant disease.

## Background

Leukaemia is a haematological malignancy which may present either acutely or chronically and is broadly classified into lymphocytic and myelogenous types. Chronic leukaemias typically progress slowly, unlike acute leukaemias, which require immediate treatment [1].

Acute lymphoblastic leukaemia (ALL) is commonly treated with a combination of chemotherapeutic drugs (such as vincristine and asparaginase) and steroids with high dose dexamethasone used to overcome drug resistance in T cell ALL and high-risk ALL [2]. Acute myeloid leukaemia (AML), however, is not yet targeted by steroids. We investigate ongoing research towards this goal. Medically used steroids include glucocorticoids, mineralocorticoids, and sex steroids. As well as naturally occurring steroids, synthetic versions of all of these have been developed [3]. The corticosteroids being discussed in this literature review are prednisone and dexamethasone, both being synthetic glucocorticoids.

Due to the high tendency for leukaemic blasts to infiltrate the cerebrospinal fluid (CSF) in ALL, certain earlier treatment protocols for patients at high risk of central nervous system (CNS) disease included the use of prophylactic cranial irradiation. Since irradiation to the CNS has a wide range of acute and chronic complications, a combination of chemotherapeutic drugs (such as cytarabine and methotrexate) and glucocorticoids may be administered intrathecally instead [4]. In fact, prophylactic cranial irradiation has nowadays been effectively replaced with chemotherapy and dexamethasone [5].

Clinical trials comparing prednisone and dexamethasone revealed that dexamethasone achieved better CNS control and less CNS relapse [6]. However, different studies report more neuropsychological and general toxicity with dexamethasone and greater incidence of death during induction, in patients receiving dexamethasone compared to prednisone [2]. Incidence of infections in patients receiving dexamethasone was also increased compared to those receiving prednisone [2, 7].

A case-controlled study in acute T cell leukaemia (ATL) patients being treated with chemotherapy alone, chemotherapy and steroids, or steroids alone recommended that they should also receive prophylactic treatment for the fungus, *Pneumocystis jiroveci*, known to cause Pneumocystis pneumonia [8]*.* Infections may also occur due to adrenal insufficiency secondary to prolonged steroid use, also increasing ALL mortality. Thus, certain studies recommend tapering off steroid dosage, particularly with dexamethasone [9]. Genetic variation may also affect the extent of adrenal suppression by exogenous steroids [10]. Moreover, the prolonged use of glucocorticoids may lead to glucocorticoid resistance, which is itself an adverse prognostic factor in cancer treatment [2].

Occasionally, steroid administration may lead to tumour lysis syndrome (TLS), an oncologic emergency characterised by hyperuricemia, hyperkalemia, hyperphosphatemia, and hypocalcemia due to rapid lysis of tumour cells [11, 12]. Steroid treatment may also be associated with leucocytosis [13].

An imbalance in the activation of mineralocorticoid receptors (MR) and glucocorticoid receptors (GR) in the brain seems to play a role in induced neuropsychological side effects [10]. These behavioural and mood changes may result in “steroid psychosis” [14, 15]. Interestingly, in some studies, a negative impact was also noted on the mental health of close relatives of ALL patients [15]. Severe neurobehavioural side effects with dexamethasone may be diminished by its discontinuation, or by replacement with prednisone, the simultaneous administration of hydrocortisone and even potassium supplements [10, 16, 17]. This review outlines ongoing investigation of the potential uses of steroids in the treatment of AML, including chemo-resistant disease.

## Main text

### Glucocorticoids in acute myeloid leukaemia

While glucocorticoids are established agents in the treatment of lymphoblastic leukaemia, their use in myeloid leukaemia is debatable [18]. Steroids are not included in any standard treatment protocols for AML [19]. Health professionals are reluctant to use glucocorticoids due to an increased risk of invasive fungal infections, sterile site infections, bacteraemia, sepsis, and death [18, 20]. Moreover, in vitro studies have reported that in some cases, paediatric AML cells were prone to glucocorticoid-induced proliferation as opposed to induced differentiation [21]. If extrapolated to the in vivo situation, this might increase the risk of relapse. However, the addition of dexamethasone to intensive chemotherapy results in a significant reduction in relapse and overall better survival rate in already hyperleucocytic AML patients [18]. In such cases, it is recommended that patients also receive prophylactic antifungal treatment to prevent infections.

Glucocorticoids bind to GR in the cytoplasm, which can then either homodimerise and bind DNA or remain monomeric, acting independent of DNA binding. This may alter certain transcriptomic programmes, inhibiting the activity of transcription factors (such as AP-1 and NF-κB) and/or by modulating the early inflammatory response (which is associated with chemoresistance). Thus, dexamethasone may sensitise AML stem cells to chemotherapy-induced cell death, thereby limiting the risk of leukaemic regrowth and relapse [18]. Glucocorticoids and other steroids, such as progesterone, may also exert collateral sensitivity in multidrug resistance (MDR) cell lines, which may help improve survival rates [22]. AML cells treated with dexamethasone were also shown to express higher levels of the CD38 marker after 1 week [18]. This increased CD38 expression could serve as a positive prognostic factor in treatment, as it helps the immune system to target the malignant cells. Apart from this, since the monoclonal antibody daratumumab binds to the CD38 protein, inducing malignant cell apoptosis, dexamethasone can help provide a potential new cytotoxic therapy route [23]. Moreover, with long-term dexamethasone treatment, a greater amount of monocytic CD11b/CD14-positive cells can be noted. This demonstrates that dexamethasone also encourages differentiation of AML cells, thus reducing the proliferating leukaemia cell burden [18].

Corticosteroids have already been used to treat certain complications that may be encountered during the therapy of AML. In acute promyelocytic leukaemia (APL), steroids are used in patients who develop differentiation syndrome, an unpredictable, but frequent complication of all-trans retinoic acid (ATRA) administration [24].

### Cardiotonic steroids

Cardiotonic steroids (CS) are natural compounds which are found in both the animal and plant kingdoms. They have long been used in the medical field to treat heart-related problems, namely heart failure and arrhythmias [22]. The cytotoxic properties of CS were first recognised when higher plasma concentrations of digitoxin were correlated with lower risks of leukaemia and urinary tract cancers [25]. A number of studies were then conducted regarding the effects that CS have on malignant cells. These drugs are capable of either binding to Na^+^/K^+^ ATPase pump [22] or inhibiting the ATP-binding cassette sub-family B member 1 (ABCB1) transporter (also known as P-glycoprotein) [25].

Genetic and epigenetic differences amongst various cancer cells lead to the acquisition of drug resistance [26]. Mechanisms of cancer resistance include increased efflux of the drug from the cell and decreased cytosolic intake of the drug. Other mechanisms include the inhibition of the apoptotic pathways, as well as the activation of cellular growth and DNA repair pathways. MDR cell lines are capable of keeping intracellular drug concentrations at very low levels by different mechanisms. Such MDR cell lines have enhanced expression of ABCB1 transporters, which pump out chemotherapeutic drugs, including anthracyclines, taxanes, and vinca alkaloids [22, 27]. Inhibition of P-glycoprotein results in inhibition of MDR, which leads to an overall better prognosis when treating concomitantly with chemotherapy [25].

MDR cells have also altered expression of other membrane proteins, including the Na^+^/ K^+^ ATPase pumps and Na^+^/H^+^ exchanger. MDR cell lines typically have downregulation of Na^+^/K^+^ ATPase pumps and upregulation of the Na^+^/H^+^ exchangers. The latter results in an increase in cellular pH, which is typically seen in malignant cells. Moreover, downstream signalling pathways associated with the Na^+^/K^+^ ATPase pumps are also commonly deregulated. The administration of correct doses of CS, such as ouabain and digitalis, will reverse MDR and increase intracellular acidity, which suppresses cancer cell growth [22].

Interestingly, the most potent CS compounds induce a greater cytotoxic effect on resistant cell lines than on normal, non-malignant white blood cells [22]. It is hypothesised that CS induce apoptosis in malignant cells through the phenomenon of collateral sensitivity due to P-glycoprotein inhibition. Collateral inhibition explains how a particular drug may exert greater cytotoxic effects in more resistant leukaemic cell lines compared to other cells. For example, with verapamil (a P-glycoprotein inhibitor), the rate at which this drug is removed from the cell by P-glycoprotein in MDR cell lines is much faster than in non-MDR cell lines. Pumping out verapamil from within the cell consumes high amounts of adenosine triphosphate (ATP), which has to be re-supplied via oxidative phosphorylation. This in turn generates high amounts of reactive oxygen species (ROS), which eventually leads to apoptosis [22].

A number of CS/CS derivatives have shown overall promising results, attributed to their low-resistance indices, when compared to the resistance indices of other established chemotherapeutic agents. The resistance index is a mathematical calculation which takes into consideration the respective half maximal inhibitory concentration (IC_50_) value of each drug in the resistant cells and the sensitive ones [22, 25]. Therefore, CS may possibly be considered in patients with leukaemias resistant to multiple chemotherapeutic drugs.

To date, clinical trials have been carried out to examine the effectiveness of CS on solid tumours, where mixed results were obtained [25]. In leukaemia, only in vitro studies have been carried out so far. These have shown promising results, but clinical trials are essential to establish their suitability for inclusion in potential future treatment regimens [22, 25].

### Ecdysteroids

Ecdysteroids are a family of natural compounds, found in different organisms, including insects, other arthropods, and plants. In insects, they play a key hormonal role in moulting and development. In plants, they serve as protection from phytophagous animals. They have also been identified in fungal extracts, but their role there has not yet been deduced [28]. It is hypothesised that these compounds are unable to interact with the mammalian steroid-hormone receptors due to fundamental differences in their structure, polarity, bulk, and shape [29].

Despite this, the addition of ecdysteroids/ecdysteroid derivatives, namely 20-hydroxyecdysone; 20-hydroxyecdysone 20,22-acetonide; 20-hydroxyecdysone 2,3;20,22-diacetonide, with various chemotherapeutic agents, has yielded better results in vitro overall [28]. These ecdysteroids/ecdysteroid derivatives are known inhibitors of the ABCB1 transporter (similar to CS [25]). However, besides yielding positive results on MDR-cell lines, the least polar ecdysteroid derivatives also showed positive outcomes on non-MDR cell lines. The IC_50_ values of the chemotherapeutic agents (doxorubicin, paclitaxel, and vincristine) decreased considerably when used simultaneously with ecdysteroids/ecdysteroid derivatives. On the other hand, when used with cisplatin, these same ecdysteroids/ecdysteroid derivatives caused an increase in the IC_50_ of the same malignant cells. This indicates that collaborative cytotoxicity does not always occur and is probably dependent on the mechanism of action of the particular chemotherapy, with alkylating-like agents being rendered paradoxically less efficient.

Other studies suggest that the ecdysteroid, β-ecdysone (20-hydroxyecdysone) derived from *Helleborus niger* (a traditional medicinal plant native to large parts of Europe), targets the mitochondrial outer membrane permeabilisation pathway (MOMP), modulating the mechanism of apoptosis. The BCL-2 family of proteins are regulators of apoptosis. It was noted that β-ecdysone (20-hydroxyecdysone) counters the MOMP inhibition pathway mediated by the anti-apoptotic BCL-2 protein. Moreover, 20-hydroxyecdysone also stimulates CD2 presentation on T-lymphocytes and aids in the function of the immune system. Since *Helleborus niger* has other active components besides β-ecdysone (20-Hydroxyecdysone), it cannot be concluded that the plant extracts’ apoptotic properties are solely the result of ecdysteroids and this requires further studies [30].

In contrast to their pro-apoptotic effect on leukaemic cells [30], ecdysteroids exert an anti-apoptotic effect on non-cancerous cell lines. In this context, most refer to the phosphatidylinositol-3-kinase/protein kinase B signal transduction (PI3K/Akt) pathway, which is responsible for the anti-apoptotic effects of ecdysteroids [29, 31]. Thus, ecdysteroids appear to specifically target neoplastic cells and may yet be developed as an additional tool in the clinical armamentarium.

To date, only in vitro studies of ecdysteroids have been conducted in AML. Based on these encouraging results, it is hoped that clinical trials will be developed in the near future.

## Conclusion

Glucocorticoids already play a major role in the treatment of ALL but are being shown to be useful in certain niche areas of treatment of AML. AML with hyperleucocytosis or resistant to classical chemotherapy are some of these. Glucocorticoids may also play a role in inducing differentiation, making cells more susceptible to immune targeting. Less classically recognised steroids, such as CS and ecdysteroids, also appear to enhance the cytotoxicity of standard chemotherapy by inhibiting efflux pumps or via other mechanisms, but this may be agent specific in certain cases. Based on this interesting in vitro data, it is surmised that clinical trials may soon be proposed to assess whether these steroids can be used in the clinic to treat certain types of AML. More research in this area is required and to be encouraged.

## Data Availability

Not applicable

## Abbreviations

ALL: Acute lymphoblastic leukaemia
CSF: Cerebrospinal fluid
CNS: Central nervous system
GR: Glucocorticoid receptor
AP-1: Activating protein 1
NF-κB: Nuclear factor-κB
MDR: Multidrug resistant
TLS: Tumour lysis syndrome
AML: Acute myeloid leukaemia
ATL: Acute T cell leukaemia
CD38: Cluster of differentiation 38
APL: Acute promyelocytic leukaemia
ATRA: All-trans retinoic acid
CS: Cardiotonic steroid
ABCB1: ATP-binding cassette sub- family B member 1
IC_50_: Half maximal inhibitory concentration
ATP: Adenosine triphosphate
ROS: Reactive oxygen species
MOMP: Mitochondrial outer membrane permeabilisation
BCL-2: B cell lymphoma 2
CD2: Cluster of differentiation 2
PI3k/Akt: Phosphatidylinositol-3-kinase/Protein Kinase B

## References

[1] American Cancer Society: Leukemia. 2020. https://www.cancer.org/cancer/leukemia.html. Accessed 16th September 2020.

[2] Inaba H, Pui CH. Glucocorticoid use in acute lymphoblastic leukemia. Lancet Oncol. 2010;11(11):1096–106. 10.1016/S1470-2045(10)70114-5. 20947430 10.1016/S1470-2045(10)70114-5 PMC3309707

[3] Hodgens A, Sharman T. Corticosteroids. 2020 Oct 1. In: StatPearls [Internet]. Treasure Island (FL): StatPearls Publishing; 2020.

[4] Pui CH, Howard SC. Current management and challenges of malignant disease in CNS in paediatric leukaemia. Lancet Oncol. 2008;9(3):257–68. 10.1016/S1470-2045(08)70070-6. 18308251 10.1016/S1470-2045(08)70070-6

[5] Veerman AJ, Kamps WA, van den Berg H, van den Berg E, Bökkerink JP, Bruin MC, van den Heuvel-Eibrink MM, Korbijn CM, Korthof ET, van der Pal K, Stijnen T, van Weel Sipman MH, van Weerden JF, van Wering ER, van der Does-van den Berg A, Dutch Childhood Oncology Group. Dexamethasone-based therapy for childhood acute lymphoblastic leukaemia: results of the prospective Dutch Childhood Oncology Group (DCOG) protocol ALL-9 (1997-2004). Lancet Oncol. 2009;10(10):957–66. 10.1016/S1470-2045(09)70228-1. 19747876 10.1016/S1470-2045(09)70228-1

[6] Mitchell CD, Richards SM, Kinsey SE, Lilleyman J, Vora A, Eden TO. Medical Research Council Childhood Leukaemia Working Party. Benefit of dexamethasone compared with prednisolone for childhood acute lymphoblastic leukaemia: results of the UK Medical Research Council ALL97 randomized trial. Br J Haematol. 2005;129(6):734–45. 10.1111/j.1365-2141.2005.05509.x. 15952999 10.1111/j.1365-2141.2005.05509.x

[7] Vrooman LM, Neuberg DS, Stevenson KE, Supko JG, Sallan SE, Silverman LB. Dexamethasone and individualized asparaginase dosing are each associated with superior event-free survival in childhood acute lymphoblastic leukemia: results from DFCI-ALL Consortium Protocol 00-01. Blood. 2009;114(22):321. 10.1182/blood.V114.22.321.321.

[8] Maeda T, Babazono A, Nishi T, Matsuda S, Fushimi K, Fujimori K. Quantification of the effect of chemotherapy and steroids on risk of Pneumocystis jiroveci among hospitalized patients with adult T-cell leukaemia. Br J Haematol. 2015;168(4):501–6. 10.1111/bjh.13154. 25266912 10.1111/bjh.13154

[9] Saracco P, Bertorello N, Farinasso L, Einaudi S, Barisone E, Altare F, Corrias A, Pastore G. Steroid withdrawal syndrome during steroid tapering in childhood acute lymphoblastic leukemia: a controlled study comparing prednisone versus dexamethasone in induction phase. J Pediatr Hematol Oncol. 2005 Mar;27(3):141–4. 10.1097/01.mph.0000155870.38794.e7. 15750445 10.1097/01.mph.0000155870.38794.e7

[10] Warris LT, van den Akker EL, Aarsen FK, Bierings MB, van den Bos C, Tissing WJ, Sassen SD, Veening MA, Zwaan CM, Pieters R, van den Heuvel-Eibrink MM. Predicting the neurobehavioral side effects of dexamethasone in pediatric acute lymphoblastic leukemia. Psychoneuroendocrinology. 2016;72:190–5. 10.1016/j.psyneuen.2016.07.006. 27448086 10.1016/j.psyneuen.2016.07.006

[11] Belay Y, Yirdaw K, Enawgaw B. Tumor lysis syndrome in patients with hematological malignancies. J Oncol. 2017;2017:9684909. 10.1155/2017/9684909. 29230244 10.1155/2017/9684909 PMC5688348

[12] Gupta A, Moore JA. Tumor lysis syndrome. JAMA Oncol. 2018;4(6):895. 10.1001/jamaoncol.2018.0613. 29801143 10.1001/jamaoncol.2018.0613

[13] Riley LK, Rupert J. Evaluation of patients with leukocytosis. Am Fam Physician. 2015;92(11):1004–11. 26760415

[14] Danilczuk Z, Ossowska G, Lupina T, Cieślik K, Zebrowska-Łupina I. Effect of NMDA receptor antagonists on behavioral impairment induced by chronic treatment with dexamethasone. Pharmacol Rep. 2005;57(1):47–54. 15849376

[15] Adams M, Robling M, Grainger J, Tomlins J, Johnson A, Morris S, Velangi M, Jenney M. Quality of life Evaluation in patients receiving Steroids (the QuESt tool): initial development in children and young people with acute lymphoblastic leukaemia. Arch Dis Child. 2016;101(3):241–6. 10.1136/archdischild-2015-309139. 26699534 10.1136/archdischild-2015-309139

[16] Igarashi S, Manabe A, Ohara A, Kumagai M, Saito T, Okimoto Y, Kamijo T, Isoyama K, Kajiwara M, Sotomatsu M, Sugita K, Sugita K, Maeda M, Yabe H, Kinoshita A, Kaneko T, Hayashi Y, Ikuta K, Hanada R, Tsuchida M. No advantage of dexamethasone over prednisolone for the outcome of standard- and intermediate-risk childhood acute lymphoblastic leukemia in the Tokyo Children's Cancer Study Group L95-14 protocol. J Clin Oncol. 2005;23(27):6489–98. 10.1200/JCO.2005.01.982. 16170158 10.1200/JCO.2005.01.982

[17] Bostrom BC, Sensel MR, Sather HN, Gaynon PS, La MK, Johnston K, Erdmann GR, Gold S, Heerema NA, Hutchinson RJ, Provisor AJ. Trigg ME; Children's Cancer Group. Dexamethasone versus prednisone and daily oral versus weekly intravenous mercaptopurine for patients with standard-risk acute lymphoblastic leukemia: a report from the Children’s Cancer Group. Blood. 2003;101(10):3809–17. 10.1182/blood-2002-08-2454. 12531809 10.1182/blood-2002-08-2454

[18] Bertoli S, Picard M, Bérard E, Griessinger E, Larrue C, Mouchel PL, Vergez F, Tavitian S, Yon E, Ruiz J, Delabesse E, Luquet I, Linares LK, Saland E, Caroll M. Danet- Desnoyers G, Sarry A, Huguet F, Sarry JE. Dexamethasone in hyperleukocytic acute myeloid leukemia. Haematologica. 2018;103(6):988–98. 10.3324/haematol.2017.184267. 29519869 10.3324/haematol.2017.184267 PMC6058767

[19] American Cancer Society: Typical treatment of acute myeloid leukemia (except APL). 2020. https://www.cancer.org/cancer/acute-myeloid-leukemia/treating/typical-treatment-of-aml.html. Accessed 13^th^ September 2020.

[20] Dix D, Cellot S, Price V, Gillmeister B, Ethier MC, Johnston DL, Lewis V, Michon B, Mitchell D, Stobart K, Yanofsky R, Portwine C, Silva M, Bowes L, Zelcer S, Brossard J, Traubici J, Allen U, Beyene J, Sung L. Association between corticosteroids and infection, sepsis, and infectious death in pediatric acute myeloid leukemia (AML): results from the Canadian infections in AML research group. Clin Infect Dis. 2012 Dec;55(12):1608–14. 10.1093/cid/cis774. 22955431 10.1093/cid/cis774

[21] Klein K, Haarman EG, de Haas V, Zwaan CM, Creutzig U, Kaspers GL. Glucocorticoid-induced proliferation in untreated pediatric acute myeloid leukemic blasts. Pediatr Blood Cancer. 2016;63(8):1457–60. 10.1002/pbc.26011. 27093190 10.1002/pbc.26011

[22] Zeino M, Brenk R, Gruber L, Zehl M, Urban E, Kopp B, Efferth T. Cytotoxicity of cardiotonic steroids in sensitive and multidrug-resistant leukemia cells and the link with Na(+)/K(+)-ATPase. J Steroid Biochem Mol Biol. 2015;150:97–111. 10.1016/j.jsbmb.2015.03.008. 25797029 10.1016/j.jsbmb.2015.03.008

[23] American Cancer Society: Drug Therapy for Multiple Myeloma*.* 2020. https://www.cancer.org/cancer/multiple-myeloma/treating/chemotherapy.html. Accessed 16^th^ September 2020

[24] Patatanian E, Thompson DF. Retinoic acid syndrome: a review. J Clin Pharm Ther. 2008;33(4):331–8. 10.1111/j.1365-2710.2008.00935.x. 18613850 10.1111/j.1365-2710.2008.00935.x

[25] Zeino M, Paulsen MS, Zehl M, Urban E, Kopp B, Efferth T. Identification of new P-glycoprotein inhibitors derived from cardiotonic steroids. Biochem Pharmacol. 2015;93(1):11–24. 10.1016/j.bcp.2014.10.009. 25451686 10.1016/j.bcp.2014.10.009

[26] Gottesman MM. Mechanisms of cancer drug resistance. Annu Rev Med. 2002;53:615–27. 10.1146/annurev.med.53.082901.103929. 11818492 10.1146/annurev.med.53.082901.103929

[27] Katayama K, Noguchi K, Sugimoto Y. Regulations of P-glycoprotein/ABCB1/MDR1 in human cancer cells. New J Sci. 2014. 10.1155/2014/476974.

[28] Martins A, Sipos P, Dér K, Csábi J, Miklos W, Berger W, Zalatnai A, Amaral L, Molnár J, Szabó-Révész P, Hunyadi A. Ecdysteroids sensitize MDR and non-MDR cancer cell lines to doxorubicin, paclitaxel, and vincristine but tend to protect them from cisplatin. Biomed Res Int. 2015;2015:895360. 10.1155/2015/895360. 26075272 10.1155/2015/895360 PMC4449901

[29] Dinan L, Lafont R. Effects and applications of arthropod steroid hormones (ecdysteroids) in mammals. J Endocrinol. 2006;191(1):1–8. 10.1677/joe.1.06900. 17065383 10.1677/joe.1.06900

[30] Jesse P, Mottke G, Eberle J, Seifert G, Henze G, Prokop A. Apoptosis-inducing activity of Helleborus niger in ALL and AML. Pediatr Blood Cancer. 2009;52(4):464–9. 10.1002/pbc.21905. 19090543 10.1002/pbc.21905

[31] Lafont R, Dinan L. Practical uses for ecdysteroids in mammals including humans: an update. J Insect Sci. 2003;3:7. 10.1093/jis/3.1.7. 15844229 10.1093/jis/3.1.7 PMC524647

